# Monitoring of health inequalities to improve health equity

**DOI:** 10.2471/BLT.24.293053

**Published:** 2025-04-01

**Authors:** Nicole Bergen, Katherine Kirkby, Devaki Nambiar, Anne Schlotheuber, Ahmad Reza Hosseinpoor

**Affiliations:** a Department of Data and Analytics, World Health Organization, Avenue Appia 20, 1211 Geneva 27, Switzerland.

Health equity – the absence of unfair, avoidable or remediable differences in health among population subgroups^1^ – is a shared goal across health and development initiatives globally. The United Nations *Transforming our world: the 2030 agenda for sustainable development* includes sustainable development goals (SDGs) devoted to ensuring healthy lives, promoting well-being for all at all ages (SDG 3) and reducing inequalities within and across countries (SDG 10).^2^ Equity is a key principle for various global health organizations^3^^–^^5^ and is a goal of many national health agendas.

Efforts to advance health equity benefit from diverse forms of evidence, including metrics on health inequalities.^1^ Understanding where health gaps exist in a population and how they are changing over time can help to inform policy-making, planning and resource allocation, and is fundamental to advancing universal health coverage.

Health inequality monitoring entails routinely quantifying and assessing health inequalities in a defined population; it uses health data from subgroups that are defined based on an association with social advantage and disadvantage. Data are analysed and reported, and the results are used alongside other forms of evidence to advance health equity.^6^ For example, countries have used inequality data to target vaccine delivery to unvaccinated children.^7^

The prerequisites for health inequality analyses include high-quality and relevant disaggregated data and analytical expertise. The inequality monitoring process, however, relies on engagement and collaboration across affected populations and other stakeholders; strong health information systems and data collection practices; developed capacities for understanding inequality analyses and interpreting results alongside other forms of evidence; effective communication of key messages to target audiences; and political will and support for remedial action and follow-up. Some of the common barriers to health inequality monitoring stem from poor data availability, weak technical capacity and lack of political will.^1^^,^^8^ As many health equity goals and ambitions are yet to be realized, increased efforts are needed to strengthen capacity for health inequality monitoring and enhance the availability and use of evidence to advance health equity. Moreover, identifying and tackling health inequalities is key to closing gaps and driving overall improvements to national averages, which are metrics that can conceal inequalities within countries. 

The Health Inequality Monitoring team at the World Health Organization (WHO) has a mandate to strengthen health inequality monitoring capacity at country, regional and global levels.^9^ To this end, WHO offers a variety of self-paced free online courses and blended learning programmes to support health inequality monitoring.^10^ The 2024 WHO book *Health inequality monitoring: harnessing data to advance health equity*^1^ consolidates foundational and emerging knowledge on health inequality monitoring. The book includes background information about the importance of inequality monitoring and how it can generate impact as well as practical information about data sources, data analysis, interpretation and reporting. The book is a key resource for capacity-building, for implementing health inequality monitoring practices and for promoting the impact of health inequality analysis.

The WHO Health Inequality Monitor website^10^ hosts other tools and resources for health inequality monitoring, including statistical codes for data disaggregation and analysis, workbooks, step-by-step manuals and templates for data source mapping. The website contains the Health Equity Assessment Toolkit, a software application that facilitates the exploration, analysis and reporting of health inequalities,^11^ and the Health Inequality Data Repository, the largest global data repository of disaggregated health data.^12^ WHO state of inequality reports are examples of technical reports based on health inequality monitoring analyses conducted for various health topics and contexts.^10^

Forthcoming WHO initiatives will help to better target and amplify capacity-strengthening for health inequality monitoring. The *Health inequality monitoring atlas* will catalogue the status of the resources, capacities and policies required for sustainable health inequality monitoring across Member States, which will help to inform the planning of targeted and coordinated support across global health partners. The WHO Health Inequality Monitoring Network will contribute to strengthening Member States’ capacity for the effective utilization of health inequality monitoring best practices, tools and resources, and facilitate knowledge exchange.

WHO’s efforts to support more standardized and rigorous approaches to health inequality monitoring are part of larger efforts to equitably improve population health.
